# Supplementary figures and images for: EWSR1::ATF1 Orchestrates the Clear Cell Sarcoma Transcriptome in Human Tumors and a Mouse Genetic Model
Source: Cancers (Basel). 2023 Dec 8;15(24):5750. doi: 10.3390/cancers15245750 (PMC10742207; doi:10.3390/cancers15245750)

V5

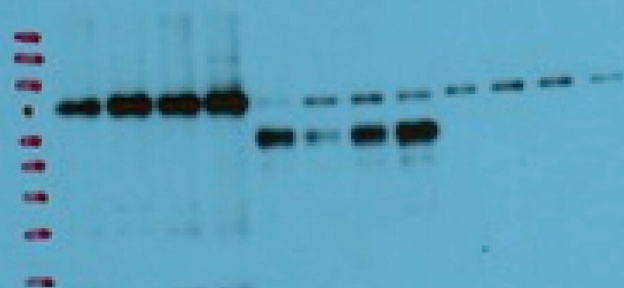

EWS

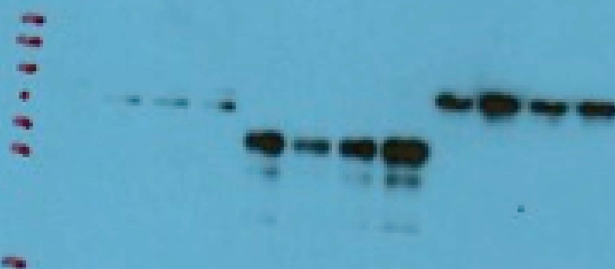

V5

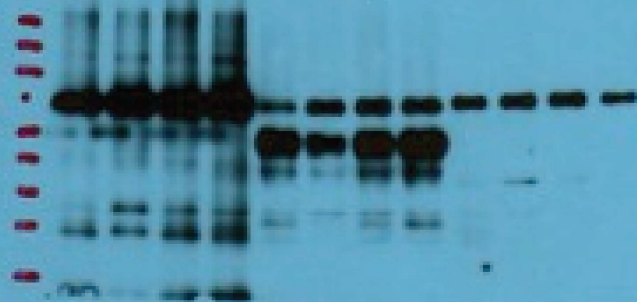

EWS

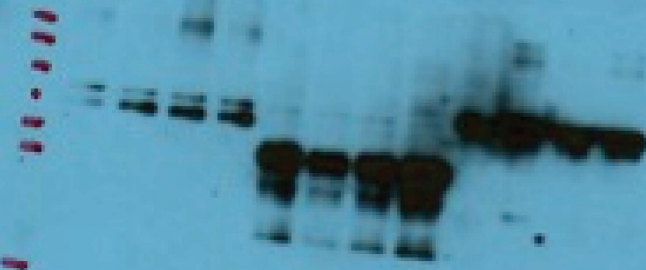

Supplement: Supplementary file 1 [file cancers-15-05750-s001.zip › cancers-2722118-Figure S1. The original immunoblot figures.pdf]
